# Supplementary material for: Skin TDP-43 pathology as a candidate biomarker for predicting amyotrophic lateral sclerosis decades prior to motor symptom onset
Source: bioRxiv. 2025 Oct 3:2025.04.10.648122. Preprint. [Version 2] doi: 10.1101/2025.04.10.648122 (PMC12621890; doi:10.1101/2025.04.10.648122)
Supplement: Supplement 1 [file NIHPP2025.04.10.648122v2-supplement-1.pdf]

bioRxiv preprint doi: https://doi.org/10.1101/2025.04.10.648122; this version posted October 3, 2025. The copyright holder for this preprint (which was not certified by peer review) is the author/funder, who has granted bioRxiv a license to display the preprint in perpetuity. It is made available under aCC-BY 4.0 International license.

Supplementary Figures

Supplementary Figure 1.

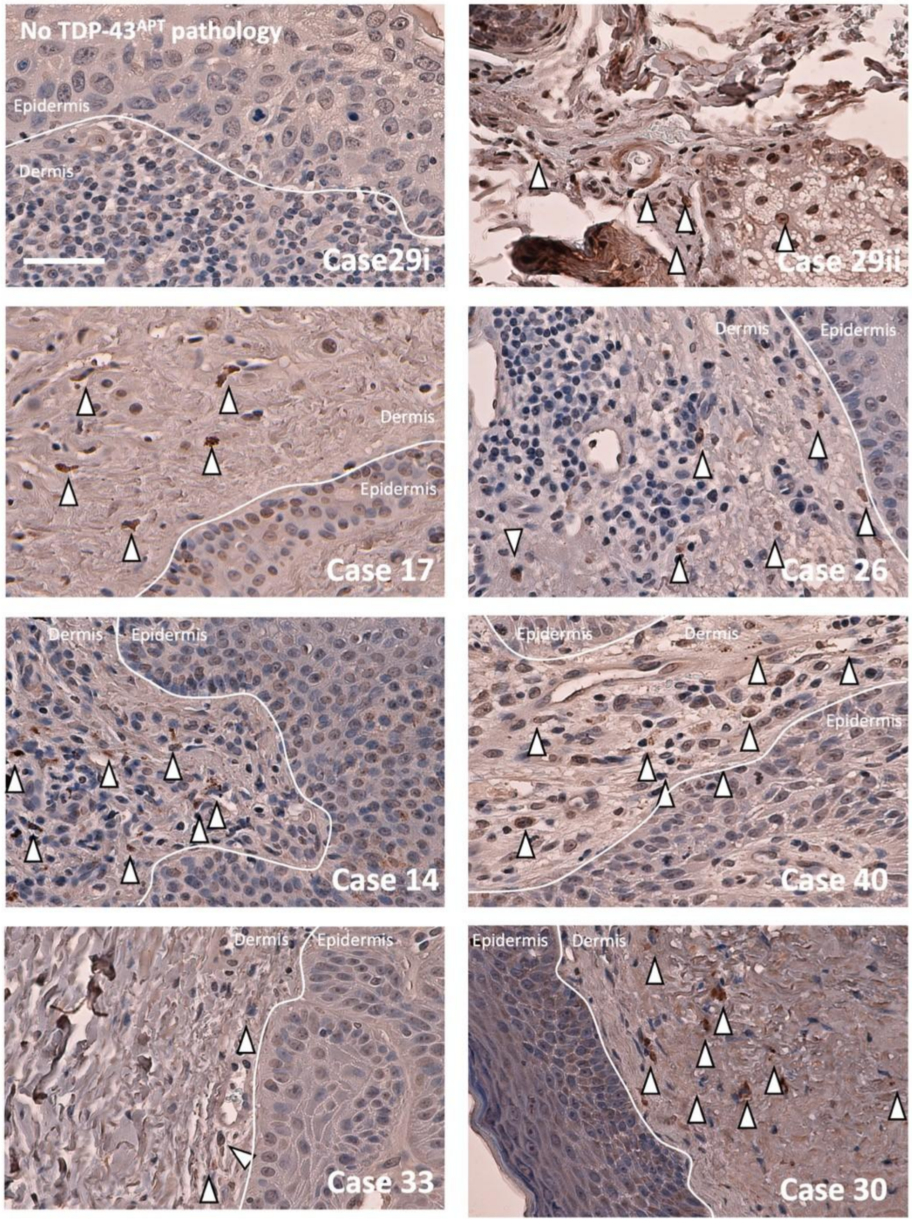

S

Supplementary Figure 1. ***TDP-43^{APT} pathology detected in all skin biopsies from individuals who went on to develop ALS.*** Photomicrographs taken at 40x of all skin biopsies from this cohort (n=7 individuals). One individual (case 29), who had a *C9orf72* mutation, had two biopsies taken 24 months apart (29i and 29ii). Sections are stained with TDP-43^{APT} (DAB chromogen in brown) and counterstained with haematoxylin (blue). TDP-43^{APT} pathology is indicated with white arrowheads and can be seen within the dendritic cells of the dermis, within neurovascular bundles, and sebaceous units. Delineation between dermis and epidermis is indicated by a continuous white line. Patient ID (case number) relates to clinical demographics published previously for this cohort (*28*). Scale bar = 20 µm.

bioRxiv preprint doi: https://doi.org/10.1101/2025.04.10.648122; this version posted October 3, 2025. The copyright holder for this preprint (which was not certified by peer review) is the author/funder, who has granted bioRxiv a license to display the preprint in perpetuity. It is made available under aCC-BY 4.0 International license.

Supplementary Figure 2.

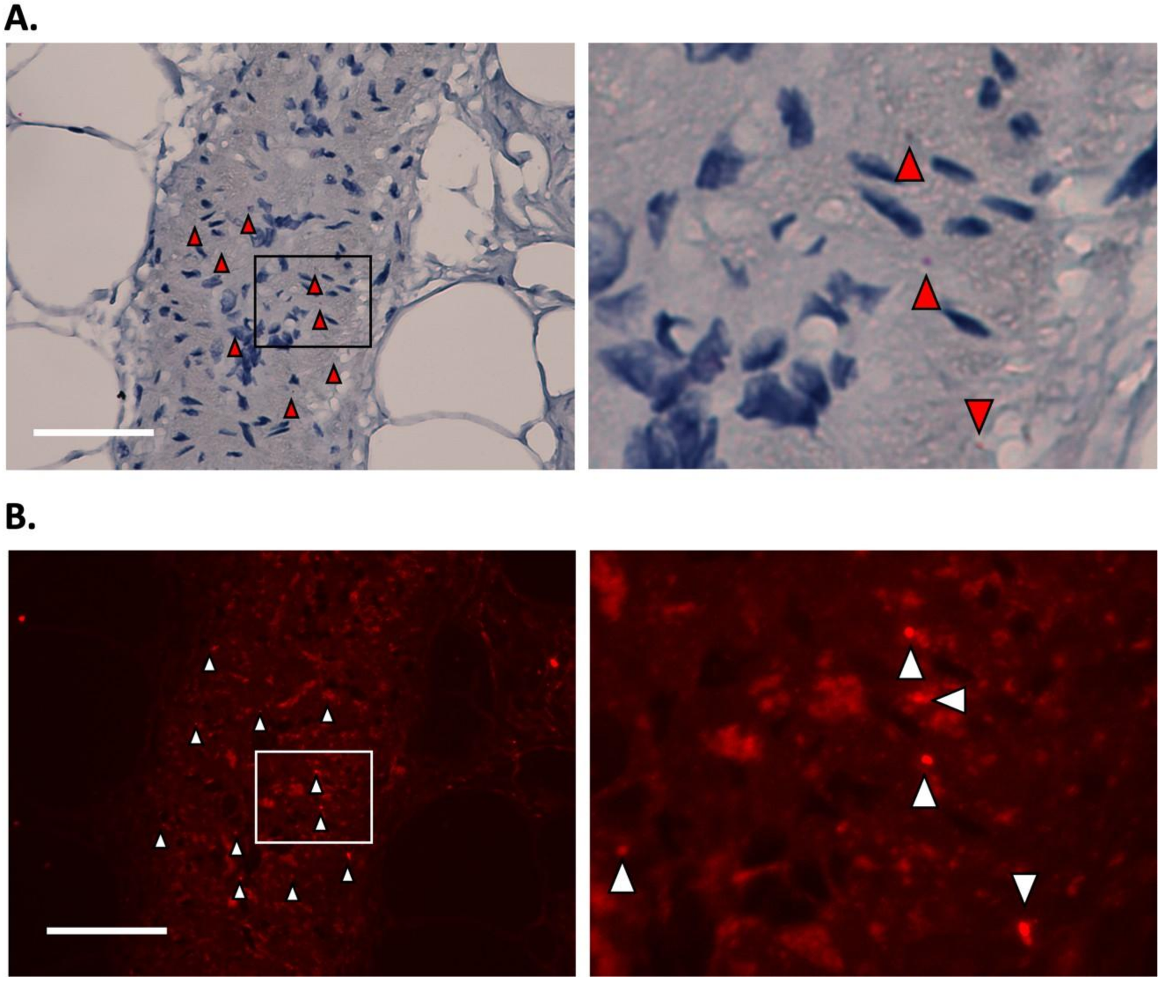

Supplementary Figure 2. ***TDP-43 pathology seen in neurovascular bundle within the fibroadipose tissue separating fascicles of muscle fibres.*** **A.** Photomicrographs taken at 20x magnification (left image), and 4 times optical zoom of the area highlighted with the black box (right image) stained with *in situ* hybridisation probes directed against the cryptic exon of STMN-2 and counterstained with haematoxylin. Signal can only be seen in the context of a loss of TDP-43 function, where it is no longer repressing cryptic exon inclusion. **B**. The same frame from (**A**) imaged using fluorescence microscopy (fast red chromogen is also a fluorophore), highlighting individual mRNA molecules of STMN-2 transcripts that contain cryptic exons indicating TDP-43 loss-of-function. Scale bar = 50 µm.

bioRxiv preprint doi: https://doi.org/10.1101/2025.04.10.648122; this version posted October 3, 2025. The copyright holder for this preprint (which was not certified by peer review) is the author/funder, who has granted bioRxiv a license to display the preprint in perpetuity. It is made available under aCC-BY 4.0 International license.

Supplementary Figure 3.

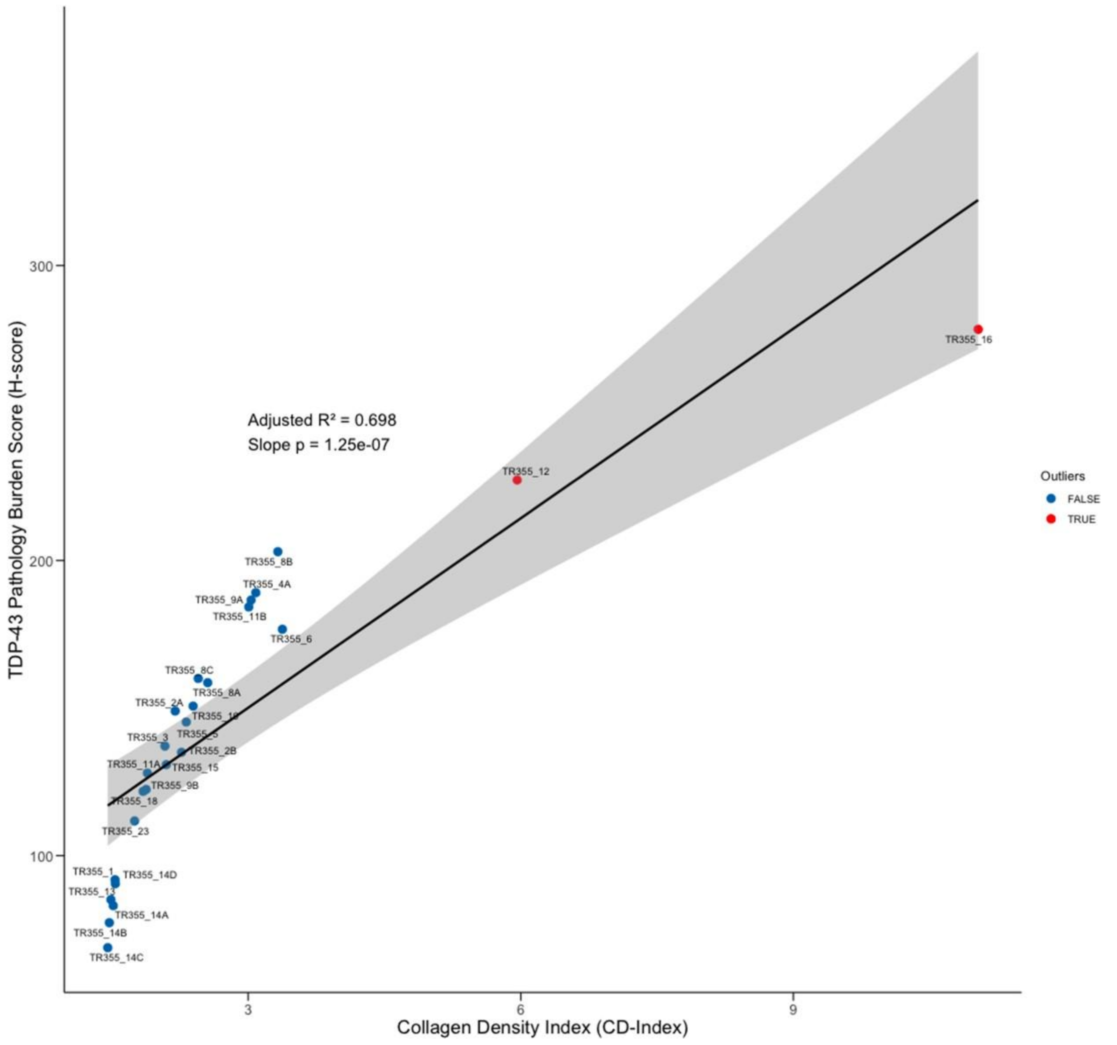

Supplementary Figure 3. Skin collagen density shows a very strong linear relationship with TDP-43 pathology, robust to the inclusion of outlier samples TR355_12 and TR355_16. Scatterplot illustrating the strong linear relationship between skin collagen density index (CD-index) and TDP-43 pathology burden amongst skin biopsies when two outlier samples are also included (giving n=25 biopsies from 17 individuals) taken from the validation cohort.

bioRxiv preprint doi: https://doi.org/10.1101/2025.04.10.648122; this version posted October 3, 2025. The copyright holder for this preprint (which was not certified by peer review) is the author/funder, who has granted bioRxiv a license to display the preprint in perpetuity. It is made available under aCC-BY 4.0 International license.

Supplementary Table 1. TDP-43 aptamer and STMN-2 cryptic exon BaseScopeTM in situ hybridisation probe pathology detection modalities provide greater sensitivity and equivalent specificity to antibody approaches for detecting TDP-43 pathology in ALS pre-symptomatic peripheral tissues. Summary table of clinicopathological information for the biomarker discovery cohort (n = 13 individuals) which was screened for TDP-43 pathology in this study, presented along with presence or absence of TDP-43 pathology detected previously using antibody approaches (*28*). Each row lists the individual ID, ALS subtype, age at biopsy, age at death, biopsy specimen type, contemporaneous pathological diagnosis, along with the presence or absence of TDP-43 pathology. For several individuals, multiple tissue samples collected at different times or from distinct anatomical sites are presented.

Supplementary Table 1. TDP-43 aptamer and STMN-2 cryptic exon BaseScope™ *in situ* hybridisation probe pathology detection modalities provide greater sensitivity and equivalent specificity to antibody approaches for detecting TDP-43 pathology in ALS pre-symptomatic peripheral tissues

Individual ID	ALS Type	Age at biopsy	Age at death	Specimen type	Diagnosis	TDP-43 Pathology	
						pTDP-43 *1	TDP-43 RNA aptamer
1	sALS	62	65	Colon	Normal	Present	Present
		62		Muscle	Denervation	Absent *2	**Present**
3	sALS	53	72	Thyroid	Goitre (adenomatous)	Absent	Absent
11	sALS	45	59	Endometrium	Endometrium (secretory)	Absent	Absent
		57		Endometrium	Endometrium (inactive)	Absent	Absent
14	sALS	54	61	Skin	Seborrheic keratosis	Present	Present
17	sALS	44	56	Cervix	Polyp	Absent	Absent
		44		Skin	Seborrheic keratosis	Absent	**Present**
		46		Uterus	Leiomyoma	Absent	Absent
18	SALS	73	74	Urethra	Chronic inflammation	Absent	Absent
26	sALS	68	70	Skin	Basal cell carcinoma	Present	Present
		55		Lipoma	No malignancy	Absent *2	Absent
				Lymph node	No malignancy	Present	Present
28	sALS	74	76	Lymph node	No malignancy	Absent	**Present**
		70		Pleura	No malignancy	Absent	Absent
29*3	*C9orf72*	55	66	Skin	Squamous cell carcinoma in situ	Absent	Absent *3
		57		Skin	Hyperkeratosis	Absent	**Present *3**
30	sALS	67	73	Skin	Pigmented nevus	Absent	**Present**
		61		Uterus	Adenocarcinoma	Absent	Absent
31	sALS	75	78	Gall Bladder	Gallstone disease	Present	Present
33	sALS	83	84	Skin	Squamous cell carcinoma	Absent	**Present**
40	sALS	71	74	Skin	Actinic keratosis	Present	Present

*1 see Pattle et al., 2022
*2 These tissues showed no evidence of pTDP-43 pathology other than within blood vessel epithelial cells
*3 A possible phenoconversion event prior to ALS symptom onset was observed for individual 29 (with C9-ALS) – a first skin biopsy taken 24 months prior to diagnosis had no TDP-43 pathology, but a second skin biopsy within 12 months of ALS diagnosis showed florid TDP-43 pathology (see Supplementary Figure 1)
Instances where TDP-43 RNA aptamer demonstrated increased TDP-43 pathology detection sensitivity are in bold
